# Insights into Gut Microbiome Composition in Hidradenitis Suppurativa: A Comprehensive Examination of Dietary Habits and Environmental Influences

**DOI:** 10.3390/nu16111776

**Published:** 2024-06-05

**Authors:** Edyta Lelonek, Jacek C. Szepietowski

**Affiliations:** Department of Dermatology, Venereology, and Allergology, Wroclaw Medical University, 50-368 Wroclaw, Poland; elelonek@gmail.com

**Keywords:** gut microbiota, diet, hidradenitis suppurativa, dietary modifications, gut–skin axis

## Abstract

This study explores the relationship between dietary habits, environmental influences, and gut microbiome composition in individuals with hidradenitis suppurativa (HS), a chronic inflammatory skin condition. A cohort of 80 participants, equally divided into HS patients and healthy controls, was assessed through comprehensive questionnaires capturing demographics, dietary habits, and other health-related information. Fecal samples were collected and analyzed using next-generation sequencing to examine microbiome composition. Despite previous studies suggesting gut dysbiosis in HS, this research found no significant differences in alpha-diversity and Shannon diversity index between the groups. However, significant disparities in dietary habits were observed, with HS patients showing higher sugar and milk consumption. The study also identified a significant correlation between coffee consumption and the presence of certain bacterial genera. While the study did not reveal major differences in microbiome diversity, the findings on dietary habits and specific microbiome components suggest potential targets for therapeutic intervention. These results underscore the importance of further research into the gut–skin axis and its role in HS, aiming to enhance management strategies through dietary modifications and lifestyle interventions.

## 1. Introduction

Hidradenitis suppurativa (HS) stands as a persistent and debilitating inflammatory dermatological condition primarily affecting regions of the body characterized by apocrine glands, including the axillary, inguinal, submammary, and anogenital areas. The hallmark features of HS encompass the formation of painful nodules, abscesses, fistulae, and resultant scarring, ultimately culminating in a marked deterioration of patients’ quality of life. Despite its prevalence and significant impact, the precise pathophysiological mechanisms underlying HS remain elusive [1,2].

Recent investigations have shed light on the involvement of various cytokines and inflammatory mediators such as tumor necrosis factor-alpha (TNF-α), interleukins (IL)-1, IL-17, and IL-23 in the pathogenesis of HS. Furthermore, aberrant keratinization and immune dysregulation leading to follicular occlusion are considered pivotal factors in the initiation and progression of HS. The interplay of genetic predisposition and environmental factors, encompassing obesity, smoking, immune dysfunction, hormonal imbalances, and microbial dysbiosis, contributes to the multifaceted nature of HS etiology [3,4].

Of particular significance, obesity emerges as a significant contributor to HS pathogenesis, exacerbating disease severity through various mechanisms, including the secretion of pro-inflammatory cytokines and the physical occlusion of follicles [5]. Weight reduction interventions have demonstrated efficacy in ameliorating HS symptoms, underscoring the intricate relationship between obesity and disease progression [6].

Beyond obesity, emerging evidence highlights the influence of dietary factors on HS aggravation [5]. Certain dietary components, notably high glycemic index foods and saturated fats, have been implicated in triggering HS flares, whereas a diet rich in vegetables, fruits, lean proteins, and essential nutrients such as zinc and vitamin D appears to confer protective effects [7]. Additionally, adherence to a Mediterranean diet, characterized by its anti-inflammatory properties, shows promise in mitigating systemic inflammation associated with HS [8]. 

Physical activity represents another modifiable factor potentially impacting HS severity. Exercise, through its role in weight management and modulation of the inflammatory process, holds potential as an adjunctive therapy in HS management, although further research is warranted to elucidate its precise effects [9]. 

Moreover, emerging insights into the gut–skin axis underscore the potential influence of gut health on HS outcomes. Observations of a higher prevalence of inflammatory bowel diseases in HS patients and promising outcomes following dietary interventions targeting gut health highlight the intricate interplay between gut microbiota and systemic inflammatory conditions, including HS [10]. Despite these insights, the relationship between gut microbiome and HS is not fully understood. Current research has yet to comprehensively address the specific mechanisms by which gut dysbiosis may influence HS pathogenesis, representing a significant gap in our understanding. Furthermore, the therapeutic potential of interventions targeting gut dysbiosis in HS management remains underexplored.

Our study was designed to investigate the potential associations between HS, dietary habits, and gut microbiome composition. By elucidating these connections, we aim to contribute to the understanding of HS pathogenesis and identify novel therapeutic avenues. This study is significant as it addresses a crucial gap in the current literature, potentially guiding more effective, holistic approaches to HS management.

## 2. Materials and Methods

The study cohort comprised 80 individuals, evenly divided into two groups: 40 individuals diagnosed with HS and 40 healthy individuals serving as the control group.

Each participant was required to complete a comprehensive questionnaire, specifically designed for this study. The questionnaire captured a wide range of information, including demographics, physical characteristics (such as body mass, height, and body circumference measurements—self-reported), medical history (including accompanying diseases and treatments), supplementation, substance use (cigarettes, alcohol, drugs—the term ‘drugs’ refers to illegal substances), detailed gastrointestinal symptoms, dietary habits (throughout the last 5 years), and menstrual cycle patterns in women.

The participant selection criteria were carefully crafted to ensure the exclusion of individuals who had undergone systemic antibiotic therapy or had consumed probiotics or prebiotics within the preceding three months. Additionally, individuals following specific dietary regimens such as vegan or gluten-free diets, or those with concurrent systemic inflammatory diseases were excluded. Other factors considered in the selection process included a history of gastrointestinal tract surgery, malignancy, or current infections.

The diagnosis of HS was established based on stringent clinical criteria as outlined in prior research [11]. The primary characteristics of the participants are precisely delineated in Table 1.

Regarding sample collection procedures, fecal specimens were obtained from all enrolled subjects. Participants were instructed to abstain from concomitant medication usage or supplements during the collection period. Female participants were advised to provide samples outside of their menstruation period. These samples were then promptly transferred to OMNIgene tubes (GUT|OM-200, DNA Genotek, Ottawa, ON, Canada) to ensure preservation and stabilization of the fecal microbiota during both transportation and storage.

Subsequent to sample collection, DNA isolation was executed at the Genomed laboratory in Warsaw, Poland. Utilizing advanced next-generation sequencing (NGS) methodologies, DNA sequencing was performed following isolation. The genomic DNA was extracted using the Genomic Mini AX Stool kit (A&A Biotechnology, Gdynia, Poland) in strict adherence to the manufacturer’s protocols. Metagenomic analysis targeting bacterial and archaeal populations focused on the highly variable V3–V4 region of the 16S rRNA gene. Specific primer sequences, 341F and 785R, were employed for amplification of the targeted region and subsequent library preparation. Amplification was performed using Q5 Hot Start High-Fidelity 2X Master Mix under manufacturer-recommended conditions. Sequencing was conducted on a MiSeq instrument using paired-end (PE), 2 × 300 nt technology, with the v3 Illumina kit.

The initial data analysis was conducted using MiSeq Reporter (MSR) software v2.6, with further bioinformatic analysis carried out using the QIIME 2 software package (Caporaso Lab, San Francisco, CA, USA). This included read classification to the species level based on the Silva 138 reference sequence database, alongside sequence variant analysis facilitated by the DADA2 package. Notably, this process allowed for the extraction of unique biological sequences known as amplicon sequence variants (ASVs) [12,13,14,15,16,17,18,19,20].

Clinical assessments for participants diagnosed with HS entailed the utilization of the Hurley staging system and the International Hidradenitis Suppurativa Severity Score System (IHS4) to evaluate disease severity [21,22]. 

The statistical analyses were conducted using R software, version 4.1.3. For qualitative variables, chi-square and Fisher’s exact tests were used, while the Mann–Whitney U test was utilized for quantitative variables. 

The observed diversity (number of species present) and the Shannon diversity index (accounting for species abundance and evenness) were calculated for each group. Descriptive statistics and the Mann–Whitney U test were used to compare the groups. PERMANOVA (Permutational Multivariate Analysis of Variance), also known as the ADONIS test, was performed using Bray–Curtis distances to assess beta diversity between the study and control groups. This analysis evaluated the differences in species composition between the groups. To assess the influence of multiple factors on quantitative variables, we employed linear regression analysis. For binary variables, logistic regression analysis was used. A significance level of 0.05 was set for all statistical tests, with *p*-values below this threshold considered indicative of statistically significant associations [23].

Ethical considerations were at the forefront of our study design and execution. We adhered to the ethical principles outlined in the Declaration of Helsinki. Prior to the commencement of the study, we obtained approval from the Ethics Committee at the Medical University in Wroclaw, Poland (No. 100/2023). Furthermore, informed consent was obtained from all participants, ensuring they were fully aware of the study’s purpose and procedures before their involvement.

## 3. Results

**Figure 1:**
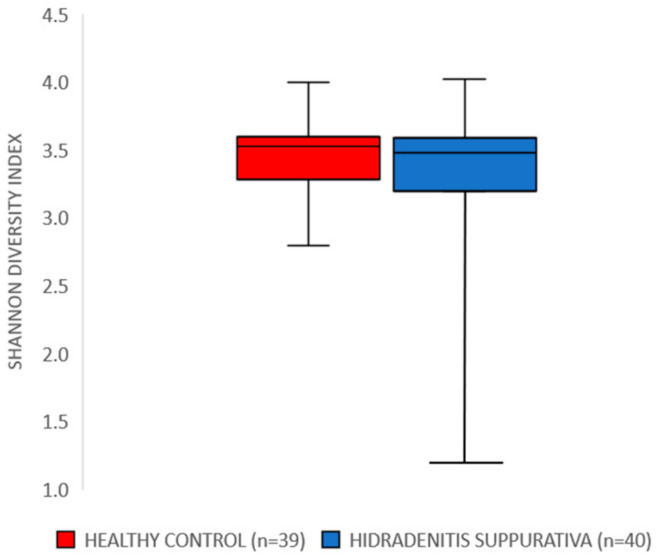
Comparison of the Shannon diversity index between the HS group and healthy volunteers.

The assessment of alpha-diversity and Shannon diversity index revealed no statistically significant differences between the HS group and healthy volunteers in terms of species richness (*p* = 0.41) and the Shannon diversity index (*p* = 0.346) (Figure 1).

**Figure 2:**
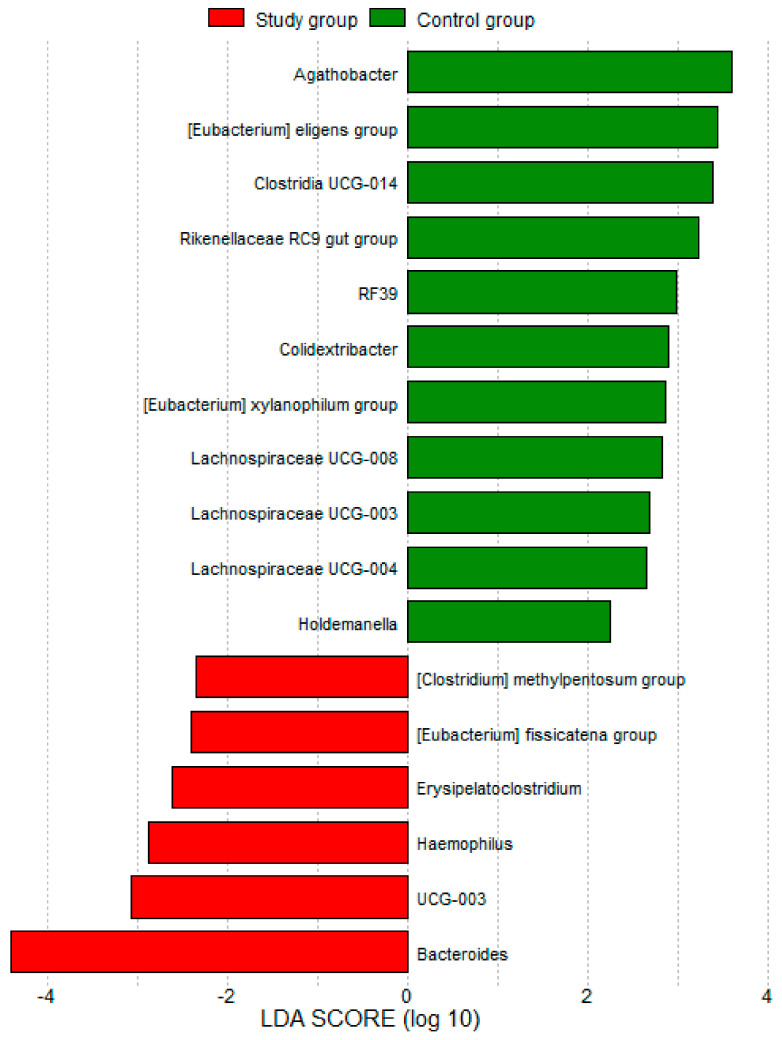
LEfSe diagram showing the significant genera in the study and control groups.

Furthermore, the comparison of gut microbiome composition using PERMANOVA statistical analyses indicated non-significant differences between the two groups (*p* > 0.05). In Figure 2, the LEfSe (Linear Discriminant Analysis Effect Size) diagram shows genera significantly associated with either the study group or the control group (LDA SCORE [log 10] < −1 and >2).

Our study investigated whether the severity of HS, as measured by the Hurley stage and the IHS4, influenced dietary adherence among patients, with adjustments for antibiotic use. The statistical analysis showed no significant association between disease severity and the likelihood of patients following a diet based on both the Hurley stage (OR = 0.851, 95% CI = 0.187–3.864, *p* = 0.834) and the IHS4 score (OR = 1.006, 95% CI = 0.965–1.048, *p* = 0.785). Moreover, the findings from multivariable logistic regression for the Hurley stage and linear regression for the IHS4 score revealed no significant effects of diet on HS severity. Specifically, the odds of progressing to Hurley Stage III were not significantly different between those on a diet and those not on a diet (OR = 0.851, 95% CI = 0.187–3.864, *p* = 0.834). Additionally, dietary adherence did not significantly affect the IHS4 score (parameter estimate = 1.325, 95% CI = −8.532–11.182, *p* = 0.794).

**Figure 3:**
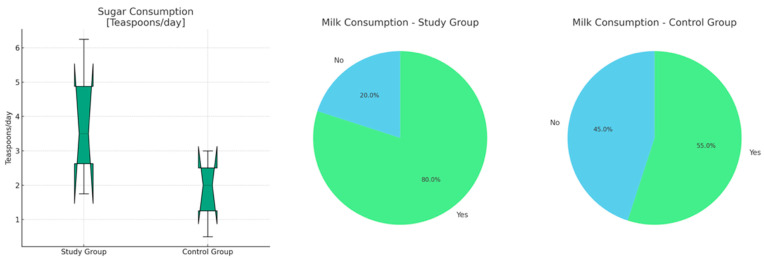
Distribution of sugar consumption and the proportion of milk consumption within studied groups.

Interestingly, our study identified significant disparities in dietary habits between the two groups. Individuals with HS exhibited notably elevated sugar consumption and a significantly heightened prevalence of milk (‘milk’ refers specifically to the consumption of milk only, excluding other dairy products such as yogurt, cheese, cream, etc.) consumption compared to the control group (*p* = 0.016 and *p* = 0.032, respectively) (Figure 3).

In our investigation focusing on the BMI of patients diagnosed with HS, logistic regression analyses were conducted separately for each genus. The results indicated that BMI significantly influences the likelihood of occurrence of several genera, as detailed in Table 2.

Furthermore, our logistic regression analysis revealed that coffee consumption significantly impacts the likelihood of occurrence of the following genera:

- *Bilophila*: OR = 3.117, suggesting that each additional cup of coffee per day increases the likelihood of *Bilophila* occurrence by 3.117 times;
- (*Ruminococcus*) *gauvreauii* group: OR = 2.274, indicating that each additional cup of coffee per day raises the likelihood of (*Ruminococcus*) *gauvreauii* group occurrence by 2.274 times;
- *UCG-003*: OR = 2.536, implying that each additional cup of coffee per day elevates the likelihood of *UCG-003* occurrence by 2.536 times.

Our linear regression analysis revealed that HS significantly influences the abundance of the following orders:

- *Desulfovibrionales*: HS increases the abundance of *Desulfovibrionales* by an average of 102.907 compared to the absence of HS;
- *Clostridia*: The regression parameter is 6.021, suggesting that HS raises the abundance of *Clostridia* by an average of 6.021 compared to the absence of HS;
- *Opitutales*: The regression parameter is −20.408, indicating that HS decreases the abundance of *Opitutales* by an average of 20.408 compared to the absence of HS.

## 4. Discussion

HS is a chronic inflammatory skin condition characterized by recurrent painful nodules, abscesses, and sinus tracts, primarily affecting areas rich in apocrine glands such as the axillae, groin, and buttocks. While the etiology of HS remains complex and multifactorial, emerging evidence suggests a potential link between dietary factors and disease severity [24]. Our study aimed to explore the association between dietary habits, gut microbiome composition, and disease severity in individuals with HS, shedding light on potential avenues for therapeutic intervention.

Several studies have compared the gut microbiome composition and diversity in HS patients and healthy controls using different methods such as culture-based techniques, 16S ribosomal RNA (rRNA) gene sequencing, and metagenomic sequencing. The results of these studies are not entirely consistent, as they have reported different degrees and directions of gut microbiome alterations in HS patients. However, some common trends can be identified, such as a lower diversity and richness of the gut microbiome, a higher abundance of pro-inflammatory bacteria, such as *Proteobacteria* and *Actinobacteria*, and a lower abundance of anti-inflammatory bacteria, such as *Firmicutes* and *Bacteroidetes*, in HS patients compared to healthy controls. These alterations may reflect a state of gut dysbiosis, which is an imbalance in the gut microbiome that can impair its functions and interactions with the host [25,26,27,28,29,30].

The mechanisms and implications of the gut microbiome alterations in HS patients are not fully elucidated, but some hypotheses can be proposed based on the existing literature. One possible mechanism is that the gut microbiome alterations in HS patients may contribute to the systemic inflammation and immune dysregulation that are characteristic of HS pathogenesis. The gut microbiome alterations may increase the production of pro-inflammatory molecules, such as lipopolysaccharide (LPS), peptidoglycan, and flagellin, that can activate the host’s PRRs and induce the secretion of pro-inflammatory cytokines, such as tumor necrosis factor-alpha (TNF-α), interleukin 1 beta (IL-1β), and interleukin 6 (IL-6), which are elevated in HS patients. The gut microbiome alterations may also decrease the production of anti-inflammatory molecules, such as short-chain fatty acids (SCFAs), that can inhibit the host’s PRRs and induce the secretion of anti-inflammatory cytokines, such as interleukin 10 (IL-10) and transforming growth factor beta (TGF-β), which are reduced in HS patients. The gut microbiome alterations may also affect the balance and function of the host’s immune cells, such as T cells, B cells, macrophages, and dendritic cells, and alter their polarization, differentiation, activation, and migration. The gut microbiome alterations may also impair the intestinal barrier function, which is the physical and immunological barrier that prevents the translocation of gut microbes and their products into the systemic circulation. The intestinal barrier function is maintained by various factors, such as tight junctions, mucus layer, antimicrobial peptides, and immunoglobulins. The gut microbiome alterations may disrupt these factors and increase intestinal permeability, which can allow the leakage of gut microbes and their products into the bloodstream and trigger systemic inflammation and immune dysregulation in HS patients [31,32].

Another possible mechanism is that the gut microbiome alterations in HS patients may influence the host’s metabolic and hormonal status, which are also involved in HS pathogenesis. The gut microbiome alterations may affect the host’s energy balance, glucose homeostasis, lipid metabolism, and insulin sensitivity, which are impaired in HS patients. The gut microbiome alterations may also affect the host’s production and regulation of various hormones, which are dysregulated in HS patients. The gut microbiome alterations may also affect the host’s skin health, as the gut microbiome can communicate with the skin microbiome, which is the collection of microorganisms and their metabolites that inhabit the skin surface. The gut microbiome alterations may alter the skin microbiome composition and function, and affect the skin’s immunity, inflammation, barrier function, and wound healing, which are impaired in HS patients [33].

The implications of gut microbiome alterations in HS patients are not only limited to the pathogenesis of HS, but also extend to the clinical manifestations, severity, and comorbidities of HS. The gut microbiome alterations may also be associated with the comorbidities of HS, such as inflammatory bowel disease, obesity, diabetes, metabolic syndrome, cardiovascular disease, depression, and anxiety, which are more prevalent in HS patients than in the general population [34,35,36].

Given the potential role of the gut microbiome in HS pathogenesis and progression, dietary interventions that can modulate the gut microbiome composition and function may be a promising strategy to improve HS symptoms and quality of life. Dietary interventions can affect the gut microbiome in various ways, such as by providing substrates for microbial fermentation, altering the pH and oxygen levels in the gut lumen, influencing the bile acid metabolism, modulating the host’s immune and metabolic responses, and introducing exogenous microorganisms or their products. Dietary interventions can be classified into different types, such as dietary patterns, dietary supplements, probiotics, prebiotics, synbiotics, and FMT [37,38].

The evidence on the effects of dietary interventions on the gut microbiome and HS symptoms is scarce and inconsistent, as there are only a few studies that have investigated this topic using different methods, populations, and outcomes. However, some preliminary findings can be summarized as follows: Dietary patterns that are high in fiber, fruits, vegetables, and plant-based foods and low in animal products, processed foods, and refined carbohydrates may be beneficial for the gut microbiome and HS symptoms, as they may increase the diversity and richness of the gut microbiome, enhance the production of SCFAs, reduce the abundance of pro-inflammatory bacteria, and lower systemic inflammation and insulin resistance in HS patients. Dietary supplements that contain omega-3 fatty acids, zinc, vitamin D, or curcumin may also be beneficial for the gut microbiome and HS symptoms, as they may modulate the gut microbiome composition and function and exert anti-inflammatory, antioxidant, and immunomodulatory effects in HS patients. Probiotics, which are live microorganisms that confer health benefits to the host, may also be beneficial for the gut microbiome and HS symptoms, as they may colonize the gut, compete with pathogenic bacteria, produce antimicrobial substances, enhance the intestinal barrier function, and modulate host immune and metabolic responses in HS patients. Prebiotics, which are non-digestible carbohydrates that selectively stimulate the growth and activity of beneficial bacteria, may also be beneficial for the gut microbiome and HS symptoms, as they may increase the abundance and diversity of the gut microbiome, promote the production of SCFAs, and improve intestinal permeability and inflammation in HS patients. Synbiotics, which are combinations of probiotics and prebiotics, may also be beneficial for the gut microbiome and HS symptoms, as they may synergistically enhance the effects of probiotics and prebiotics in HS patients. Fecal microbiota transplantation (FMT), which is the transfer of fecal matter from a healthy donor to a recipient, may also be beneficial for the gut microbiome and HS symptoms, as it may restore gut microbiome diversity and function and improve clinical outcomes in HS patients [39,40,41,42].

In our study, the alpha-diversity and Shannon diversity index of the gut microbiome showed no significant differences between individuals with HS and healthy volunteers in terms of species richness and diversity. This finding contrasts with previous studies suggesting a potential role of gut dysbiosis in the pathogenesis of HS. However, further investigations employing metagenomic sequencing and functional analyses may provide deeper insights into microbial dysregulation in HS.

Interestingly, our study identified significant disparities in dietary habits between individuals with HS and healthy controls, with the former exhibiting elevated sugar consumption and a higher prevalence of milk consumption. These findings corroborate previous research suggesting potential associations between dietary factors and HS severity. Specifically, limiting simple carbohydrate intake and dairy consumption has been proposed as dietary modifications that may alleviate HS symptoms by reducing inflammation and modulating the gut microbiome [43,44,45,46,47].

Furthermore, our regression analyses revealed significant associations between BMI and the abundance of specific bacterial genera, highlighting the potential impact of obesity on gut microbiome composition in individuals with HS. Notably, coffee consumption was found to significantly impact the likelihood of occurrence of several genera, including *Bilophila*, (*Ruminococcus*) *gauvreauii* group, and *UCG-003*. This suggests that coffee consumption may influence the gut microbiome composition in individuals with HS in different ways. *Bilophila* presence may contribute to pro-inflammatory responses and conditions like inflammatory bowel disease in some individuals [48], the (*Ruminococcus*) *gauvreauii* group can enhance systemic immune responses mediated by pro-inflammatory cytokines such as TNF-α and IL-6 h [49], whereas *UCG-003* can be associated with chronic insomnia and cardiometabolic diseases [50]. Based on these findings, it can be concluded that obesity and coffee consumption significantly influence the gut microbiome composition in individuals with HS, with specific bacterial genera being associated with potentially harmful health outcomes. This highlights the complex interactions between diet, body weight, and microbiome health.

Finally, the study revealed that HS significantly influences the abundance of several orders, including *Desulfovibrionales*, *Clostridia*, and *Opitutales*. This suggests that HS may be associated with specific alterations in the gut microbiome at the order level.

The findings of our study are consistent with existing literature emphasizing the importance of dietary modifications and lifestyle interventions in managing HS. The Mediterranean diet, characterized by its anti-inflammatory properties and emphasis on whole foods, has shown promising results in improving disease activity and quality of life in individuals with HS. Moreover, supplementation with zinc and vitamin D has been suggested as potential adjunctive therapies, although further research is warranted to elucidate their efficacy. 

One of the limitations of this study is the reliance on self-measured and self-reported data. To mitigate recall bias, we designed the questionnaire with detailed and structured prompts to assist participants in more accurately recalling their dietary habits. Despite these efforts, we acknowledge that self-reported data may still be subject to some degree of recall bias and inaccuracy.

In future studies, we plan to incorporate additional methods to validate self-reported data. These methods may include the use of dietary biomarkers or cross-referencing with food diaries maintained over shorter, more manageable periods. Such approaches will help enhance the accuracy and reliability of dietary intake data.

The directions for future research on the effects of dietary interventions on the gut microbiome and HS symptoms are manifold, as there are many gaps and challenges that need to be addressed. Some of the directions are as follows: First, more randomized controlled trials (RCTs) with larger sample sizes, longer follow-up periods, and standardized protocols are needed to confirm the efficacy and safety of dietary interventions in HS patients. Second, more mechanistic studies with advanced techniques, such as metagenomic, metatranscriptomic, metaproteomic, and metabolomic analyses, are needed to elucidate the causal and functional relationships between the gut microbiome and HS and to identify the key microbial taxa, genes, pathways, and metabolites that are involved in HS pathogenesis and progression. Third, more personalized and precise approaches are needed to tailor the dietary interventions to the individual characteristics of HS patients, such as their genetic backgrounds, disease phenotypes, gut microbiome profiles, dietary habits, and preferences. Fourth, more interdisciplinary and translational collaborations are needed to integrate knowledge and expertise from different fields, such as dermatology, gastroenterology, microbiology, nutrition, and psychology, and to translate the research findings into clinical practice and public health policy.

## 5. Conclusions

In conclusion, the gut microbiome is a complex and dynamic ecosystem that interacts with the host in various ways and influences various aspects of human health and disease, including HS. Gut microbiome alterations in HS patients may reflect a state of gut dysbiosis, which may contribute to the systemic inflammation, immune dysregulation, metabolic and hormonal imbalance, and skin impairment that are characteristic of HS pathogenesis and progression. Dietary interventions that can modulate the gut microbiome composition and function may be a promising strategy to improve HS symptoms and quality of life. However, evidence of the effects of dietary interventions on the gut microbiome and HS symptoms is scarce and inconsistent and requires further investigation. Future research should focus on conducting more RCTs, exploring more mechanistic pathways, developing more personalized and precise approaches, and fostering more interdisciplinary and translational collaborations to advance our understanding and management of HS.

## Figures and Tables

**Table 1:** Baseline characteristics of study participants.

Parameter		Group		*p*
		Study Group (*N* = 40)	Control Group (*N* = 40)	
Age (years)	mean ± SD	39.02 ± 11.69	43.23 ± 18.18	*p* = 0.494
	median	40	39.5	
	quartiles	32–46	27–60.25	
BMI (kg/m^2^)	mean ± SD	30.36 ± 6.77	25.05 ± 4.77	*p* < 0.001 *
	median	28.73	24.75	
	quartiles	25.66–32	20.59–28.83	
WHR	mean ± SD	0.87 ± 0.11	0.81 ± 0.09	*p* = 0.004 *
	median	0.86	0.81	
	quartiles	0.79–0.89	0.74–0.85	
Gender	Female	18 (45.00%)	27 (67.50%)	*p* = 0.071
	Male	22 (55.00%)	13 (32.50%)	
Residence	Rural	11 (27.50%)	8 (20.00%)	*p* = 0.599
	Urban	29 (72.50%)	32 (80.00%)	
Education	Primary	2 (5.00%)	1 (2.50%)	*p* = 0.005 *
	Vocational	8 (20.00%)	2 (5.00%)	
	Secondary	18 (45.00%)	10 (25.00%)	
	Higher	12 (30.00%)	27 (67.50%)	
Occupational status	Student	5 (12.50%)	2 (5.00%)	*p* = 0.003 *
	Employed	28 (70.00%)	30 (75.00%)	
	Unemployed	6 (15.00%)	0 (0.00%)	
	Retired	1 (2.50%)	8 (20.00%)	
Tobacco smoking	No	17 (45.50%)	33 (82.50%)	*p* = 0.001 *
	Yes	23 (57.50%)	7 (17.50%)	
Alcohol consumption	No	12 (30.00%)	10 (25.00%)	*p* = 0.773
	Yes	5 (12.50%)	7 (17.50%)	
	Occasionally	23 (57.50%)	23 (57.50%)	
Drug use	Never	29 (72.50%)	36 (90.00%)	*p* = 0.165
	Occasionally	4 (10.00%)	1 (2.50%)	
	In the past	7 (17.50%)	3 (7.50%)	
Diet	No	19 (47.50%)	23 (57.50%)	*p* = 0.502
	Yes	21 (52.50%)	17 (42.50%)	
Physical activity	Sedentary	8 (20.00%)	3 (7.50%)	*p* = 0.041 *
	Low	13 (32.50%)	11 (27.50%)	
	Moderate	13 (32.50%)	12 (30.00%)	
	Active	6 (15.00%)	7 (17.50%)	
	Very active	0 (0.00%)	7 (17.50%)	
Duration of the disease [months]	mean ± SD	93.92 ± 70.94	-	
	median	72	-	
	quartiles	54.25–99	-	
IHS4 (points)	mean ± SD	21.52 ± 16.3	-	
	median	18	-	
	quartiles	11–25.25	-	
Hurley Staging	Stage II	30 (75%)	-	
	Stage III	10 (25%)	-	

* statistically significant results.

**Table 2:** Influence of BMI on the occurrence of specific bacterial genera.

Genus	OR	95% CI		*p*
*Enterorhabdus*	0.703	0.547	0.904	0.006 *
*Senegalimassilia*	0.868	0.763	0.987	0.03 *
*Coprobacter*	0.833	0.728	0.952	0.007 *
*Gastranaerophilales*	0.863	0.763	0.976	0.019 *
*Desulfovibrio*	0.83	0.714	0.965	0.015 *
*Candidatus Stoquefichus*	0.847	0.72	0.995	0.043 *
*Erysipelatoclostridiaceae*	0.775	0.635	0.945	0.012 *
*Erysipelatoclostridium*	0.891	0.795	1	0.049 *
*Dielma*	0.765	0.607	0.963	0.023 *
*Holdemanella*	0.831	0.722	0.957	0.01 *
*Merdibacter*	1.122	1.006	1.251	0.039 *
*Solobacterium*	0.858	0.741	0.993	0.039 *
*Lactobacillus*	1.224	1.052	1.425	0.009 *
*Gemella*	1.261	1.001	1.587	0.049 *
*Christensenellaceae R-7 group*	0.874	0.769	0.993	0.039 *
*Ruminiclostridium*	0.775	0.635	0.945	0.012 *
*Clostridia vadinBB60 group*	0.835	0.731	0.954	0.008 *
*Defluviitaleaceae UCG-011*	0.839	0.733	0.961	0.012 *
*GCA-900066575*	0.89	0.797	0.993	0.037 *
*Lachnospiraceae FCS020 group*	0.878	0.782	0.986	0.028 *
*Marvinbryantia*	0.895	0.802	0.998	0.047 *
*(Eubacterium) fissicatena group*	0.814	0.684	0.969	0.021 *
*Colidextribacter*	0.818	0.686	0.974	0.024 *
*NK4A214 group*	0.858	0.756	0.975	0.019 *
*UCG-002*	0.872	0.771	0.986	0.029 *
*Anaerofilum*	0.808	0.666	0.98	0.031 *
*Anaerotruncus*	0.887	0.789	0.997	0.044 *
*Angelakisella*	0.737	0.581	0.933	0.011 *
*Candidatus Soleaferrea*	0.873	0.766	0.995	0.042 *
*DTU089*	0.867	0.768	0.979	0.021 *
*Ruminococcus*	0.869	0.764	0.989	0.033 *
*(Eubacterium) siraeum group*	0.814	0.701	0.945	0.007 *
*UCG-010*	0.863	0.766	0.973	0.016 *
*(Clostridium) methylpentosum group*	0.849	0.741	0.973	0.019 *
*Peptococcus*	0.745	0.6	0.924	0.007 *
*Family XIII UCG-001*	0.896	0.803	1	0.049 *
*DTU014*	0.864	0.757	0.987	0.031 *
*Phascolarctobacterium*	0.883	0.79	0.988	0.03 *
*Dialister*	1.126	1.008	1.257	0.035 *
*Veillonella*	1.147	1.021	1.289	0.021 *
*Comamonas*	0.737	0.582	0.934	0.011 *
*Oxalobacter*	0.852	0.746	0.973	0.018 *
*Victivallis*	0.748	0.616	0.909	0.003 *
*Akkermansia*	0.897	0.806	0.999	0.048 *

* statistically significant results.

## Data Availability

The original contributions presented in the study are included in the article, further inquiries can be directed to the corresponding author.
